# Reconstruction of nasal vestibular obstruction after total nasal reconstruction using superior subcutaneous pedicle nasolabial flaps

**DOI:** 10.1016/j.jpra.2021.04.010

**Published:** 2021-05-17

**Authors:** Motomu Suito, Takeshi Kitazawa

**Affiliations:** Department of Plastic and Reconstructive Surgery, Matsunami General Hospital, 185-1, Dendai, Kasamatsu, Hashima-gun Gifu, Japan 501-6062

**Keywords:** Nose, Nasolabial, Lining, Obstruction, Rhinoplasty, Vestibule

## Abstract

**Objective and Methods:**

Nasal obstruction after total nasal reconstruction is a serious complication that contributes to breathing difficulty, snoring, and obstructive sleep apnea, which can negatively influence daily activities. However, few treatments have been reported in detail for this condition. Here, a case of nasal vestibular obstruction after total nasal reconstruction that was treated with bilateral superior subcutaneous pedicle nasolabial flaps is reported.

**Results:**

An intranasal stent was used postoperatively for five months to prevent restenosis. Internal stenosis was not noted 25 months postoperatively. The patient could breathe easily through his nose and mouth dryness improved.

**Conclusion:**

The flap is relatively thin, easy to elevate with high flexibility and stable blood flow, and useful for nasal vestibular lining reconstruction.

## Introduction

Nasal obstruction after total nasal reconstruction is a serious complication that contributes to breathing difficulty, snoring, obstructive sleep apnea,^1^ and negatively influences daily activities. Few treatments are reported in the literature. A case of vestibular obstruction after total nasal reconstruction that was treated with bilateral superior subcutaneous pedicle nasolabial flaps (SSPNFs) is reported here.

## Case presentation

A 50-year-old man presented with nasal lobule disfigurement. He had schizophrenia and had mutilated his nose 10 years before the initial consultation. Physical examination revealed a full-thickness nasal defect, including the nasal tip, both alae nasi, caudal septum, and columella. Reconstructive surgery was scheduled because his medical condition was well-controlled and self-mutilation had not occurred for several years.

## Total nasal reconstruction

**Figure 1:**
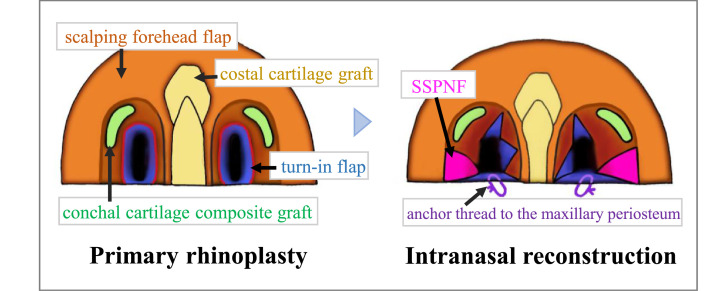
Primary rhinoplasty and Intranasal reconstruction. Primary rhinoplasty: Suture lines between the turn-in flap and scalping forehead flap were linear to surround the nasal vestibulum (the red line). Intranasal reconstruction: The SSPNFs were inserted between the scalping forehead and turn-in flaps.

Considering the nasal subunits, total nasal reconstruction was performed (Figure 1). A turn-in flap was used for the nasal vestibular lining. As a supportive structure, an L-shaped costal cartilage graft was formed and inserted in the dorsum and columella. Conchal cartilage composite grafts were inserted on both sides of the alae nasi. The outer layer was reconstructed with a scalping forehead flap. Two weeks later, the flap was divided and split-thickness skin from the thigh was grafted on the flap donor site.

**Figure 2:**
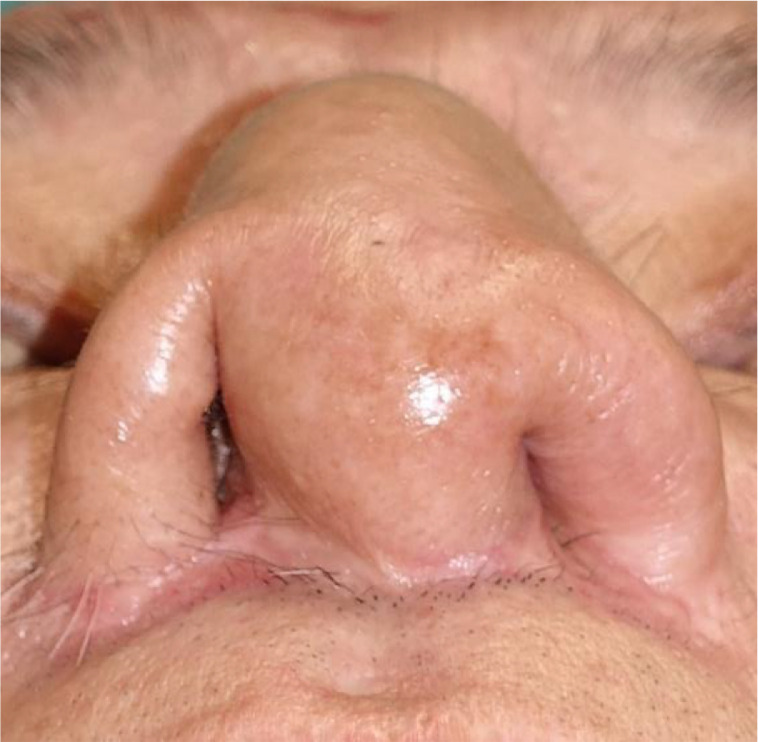
Six months after flap division. Nasal vestibular stenosis was observed.

External nasal morphology was well maintained postoperatively. However, nasal vestibular stenosis progressed, and nasal breathing became difficult (Figure 2). Therefore, we enlarged the intranasal lining six months postoperatively.

## Intranasal lining reconstruction

**Figure 3:**
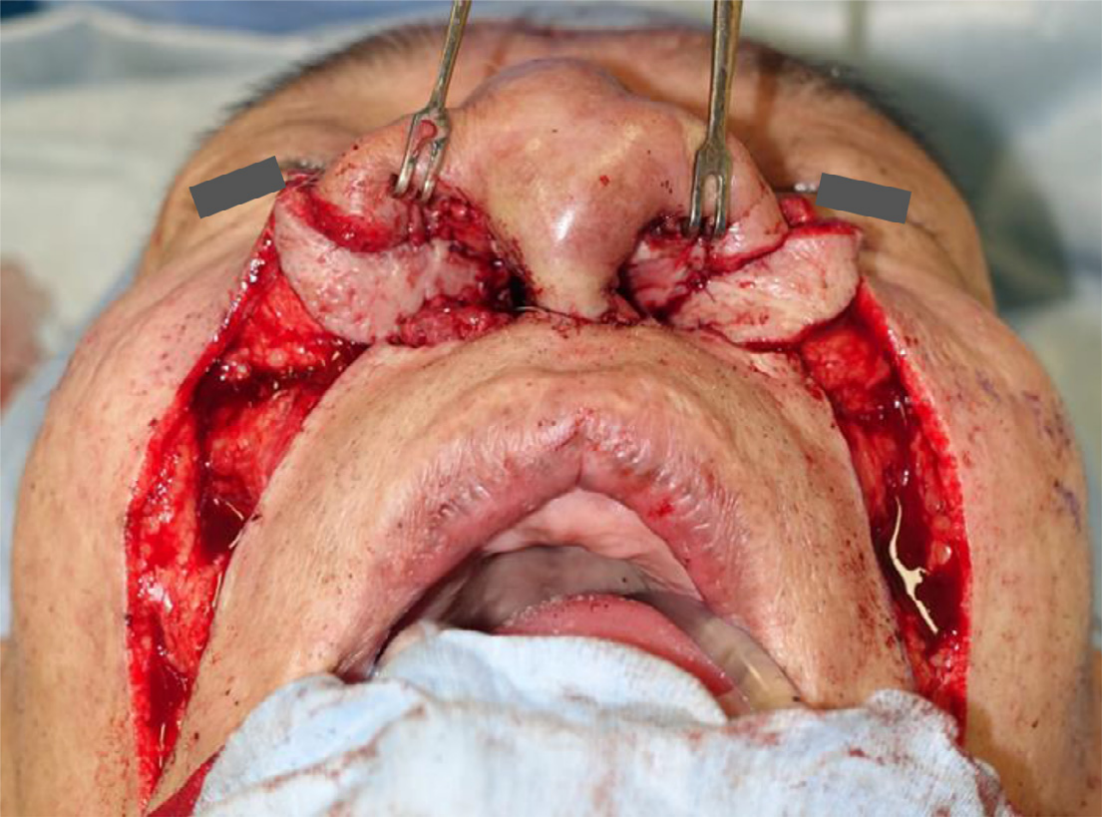
Intraoperative photograph of intranasal reconstruction. The distal position of the SSPNF was sutured to the edge of the piriform.

An incision was made between the scalping forehead and turn-in flaps, to the piriform aperture edge, and the SSPNF was inserted as follows. Nasolabial island flaps (15 × 75 mm) were designed bilaterally. The subcutaneous pedicle was 25 mm wide and 30 mm long and elevated just above the mimic muscles with dissection up to the medial eye canthus. The pivot point was located just lateral to the nasal sidewall to easily advance to the nasal vestibule. The SSPNF distal portion was excised, and the skin island (15 × 25 mm) was prepared as wide as the subcutaneous pedicle. The skin island was inserted between the scalping forehead and turn-in flaps, and its distal position was sutured to the edge of the piriform (Figure 3). The skin island at the part hidden under the skin was de-epithelialized. At the columella, the costal cartilage was shaved and the scalping forehead flap was thinned. To suppress wound contraction and enlarge the nasal vestibulum, the triangle turn-in flap was inserted into the scalping forehead flap and sutured in a zigzag fashion. To expand the nasal vestibule, the turn-in flap forming the nostril floor was anchored to the maxillary periosteum (Figure 2).

**Figure 4:**
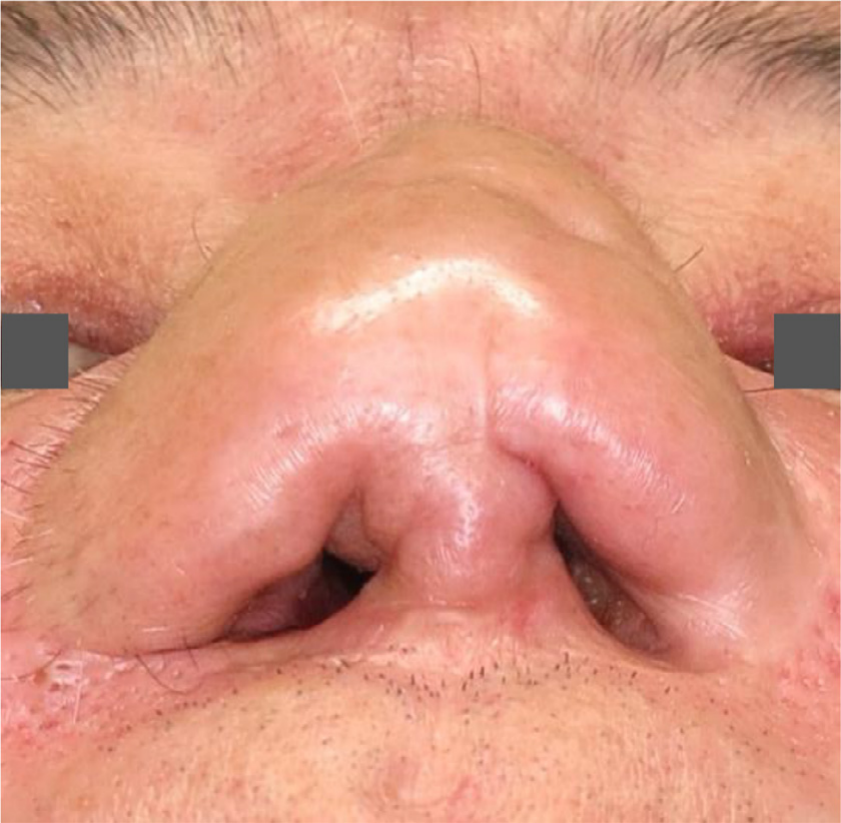
Twenty-five months after secondary reconstruction. The nasal airway was maintained.

An intranasal stent was used postoperatively for five months to prevent restenosis. The nasal airway was maintained 25 months postoperatively (Figure 4). Although it was ideal to perform additional surgery to improve the nasal morphology, the patient did not want further surgery because he was satisfied with his nasal breathing condition. His mouth dryness also improved.

## Discussion

Nasal airway obstruction after total nasal reconstruction is a serious complication causing breathing difficulty and other more serious problems. According to the Starling resistor model, nasal obstruction generates a suction force resulting in oropharyngeal collapse, thus contributing to snoring and obstructive sleep apnea,^1^ and negatively influencing daily activities.

During primary rhinoplasty, large nasal defects require trilaminar reconstruction of the external skin, structural support, and lining^2^. The intranasal lining substitute should be thin, pliable, and well-vascularized ^3^. Numerous options for the intranasal and vestibular lining include turn-in, median forehead, transverse orbicularis oculi myocutaneous, nasolabial, and septal mucosal flaps; skin grafting; and so forth^2^^,^^4^, ^5^, ^6^. Although many reports exist of simultaneous intranasal lining reconstruction during primary rhinoplasty, few have a detailed secondary reconstruction of intranasal obstruction. We enlarged the intranasal lining using SSPNFs after total nasal reconstruction. This flap was easy to elevate, relatively thin, and had high flexibility in translocation, and stable blood flow. Although the SSPNF vascular supply is random, it is stable for the wide subcutaneous pedicle, and therefore, can be relatively thinner.

The location of a nasal obstruction must be identified, the cause established, and a correction plan formulated^7^. Our main problem was nasal vestibular stenosis. The causes of the obstruction were (Figure 1): (1) thick columella; (2) the vestibule was obstructed along with wound contraction because suture lines between the turn-in and scalping forehead flaps were circumferential surrounding the nasal vestibule; and (3) the nostril floor shrank owing to wound contraction. Therefore, we performed the following treatments for the above causes (Figure 1): (1) for the thick columella, the costal cartilage was shaved and the scalping forehead flap was thinned; (2) to expand the nasal vestibule, the SSPNF was inserted to the limen nasi. The triangle flaps were inserted into the scalping forehead flap and sutured in a zigzag manner; (3) to expand the nasal vestibule, the turn-in flap that formed the nostril floor was anchored to the maxillary periosteum. The nasal airway was maintained postoperatively and significant improvement in nasal breathing was observed. Ideally, additional surgery should have been performed to improve nasal morphology.

Pre- and post-operative rhinomanometry should be performed if possible because each patient experiences the degree of nasal obstruction differently. Anterior rhinomanometry is widely used for evaluating nasal obstruction objectively^8^. In the present case, anterior rhinomanometry could not be inspected before the corrective surgery because of severe nasal obstruction. Although rhinomanometry was performed again after nasal vestibular lining reconstruction, accurate values were not available because of nasoseptal perforation created by the turn-in flap.

## Conclusion

The SSPNF is easy to elevate, relatively thin, and has high flexibility in translocation and stable blood flow. Nasoseptal perforation should be closed before anterior rhinomanometry.

## Conflict of interest

None.
